# Novel Electrochemical Approaches for Anticancer Drug Monitoring: Application of CoS@Nitrogen-Doped Amorphous Porous Carbon Composite in Nilotinib Detection

**DOI:** 10.1021/acsomega.4c05505

**Published:** 2024-12-31

**Authors:** Merve Yıldır, Asena Ayse Genc, Nesrin Buğday, Nevin Erk, Naeimeh Sadat Peighambardoust, Umut Aydemir, Sedat Yaşar

**Affiliations:** † Faculty of Pharmacy, Department of Analytical Chemistry, Ankara University, 06560 Ankara, Turkey; ‡ Graduate School of Health Sciences, Ankara University, 06110 Ankara, Turkey; § Faculty of Science and Art, Department of Chemistry, İnönü Üniversity, 44280 Malatya, Turkey; ∥ Boron and Advanced Materials Research Center (KUBAM), Koç University, 34450 Istanbul, Turkey

## Abstract

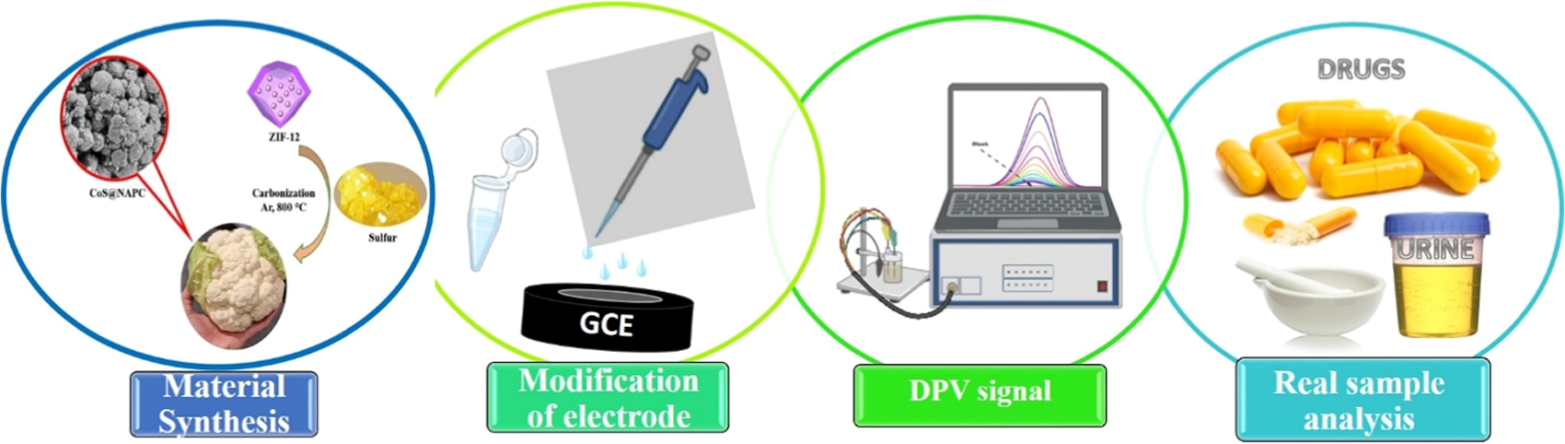

A novel composite containing CoS and nitrogen-doped amorphous porous carbon (NAPC), denoted as CoS@NAPC, was successfully synthesized from a mixture of cobalt-based ZIF-12 and sulfur through one-pot pyrolysis. The morphology and microstructure of the composites are evaluated with appropriate spectroscopic techniques. CoS@NAPC was used to modify the glassy carbon electrode (GCE) to detect Nilotinib. Under optimized conditions, the GCE electrode modified with CoS@NAPC showed a low limit of detection (LOD) of 11.8 ng/mL and two wide linear concentration ranges of 59.7–1570 and 1570–11200 ng/mL for the determination of Nilotinib. GCE electrode modified with CoS@NAPC has excellent recoveries ranging from 98.41 to 102.03% for real samples, demonstrating its superior analytical performance. The enhanced performance of CoS@NAPC as an electrochemical sensor may stem from the combined action of two key components: CoS, known for its potent reactivity toward Nilotinib, and NAPC, which offers a consistent conductive carbon matrix. CoS contributes to its reactivity, while NAPC facilitates electron transfer during Nilotinib detection. This synergistic effect likely underlies the superior sensor performance observed.

## 1 Introduction

Nilotinib (Tasigna, Novartis Pharmaceuticals), sanctioned by the United States Food and Drug Administration (FDA) in 2007, is a second-generation tyrosine kinase inhibitor (TKI) meticulously engineered to antagonize aberrant BCR-ABL (Breakpoint Cluster Region-Abelson) fusion protein.^1−3^ Nilotinib, prescribed for specific forms of chronic myelogenous leukemia (CML) associated with the Philadelphia chromosome, demonstrates 30-fold greater effectiveness against CML cells compared to Imatinib. Nilotinib exhibits superior binding affinity and selectivity for the BCR-ABL kinase in comparison to Imatinib. Moreover, it displays in vitro activity against numerous Imatinib-resistant mutants.^4,5^ Besides all these, some research found that Parkinson’s disease (PD) models and patient brain tissues had higher levels of cellular Abelson (c-Abl) activation.^6−8^ In animal models of neurodegeneration, low doses of nilotinib promote the degradation of tau and Aβ by crossing the blood–brain barrier. Clinical research indicates that nilotinib enters the central nervous system, increases dopamine turnover, and decreases tau levels in cerebrospinal fluid (CSF) independently of Abelson inhibition.^9^

**Figure 1:**
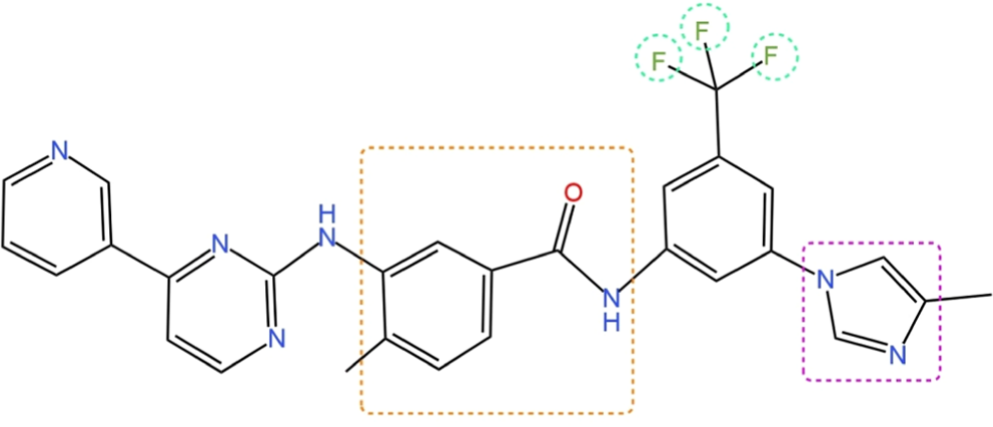
Chemical structure of Nilotinib.

Nilotinib, a white to slightly yellowish to slightly greenish-yellow powder with molecular formula C_28_H_22_F_3_N_7_O and molecular weight 529, is chemically known as 4-methyl-N-[3-(4-methyl-1-imidazol-1-yl)-5-(trifluoromethyl)phenyl]-3-benzamide (Figure 1).^10,11^ At a temperature of 25 °C, the water solubility of nilotinib (NILO) experiences a notable decline as the pH increases. Additionally, it demonstrates almost negligible solubility in buffer solutions characterized by pH values exceeding 4.5. Nilotinib exhibits slight solubility in both ethanol and methanol.^12^

Prolonged administration of nilotinib reveals distinct adverse effects, notably cardiovascular toxicity, which differ from those typically associated with other BCR/ABL tyrosine kinase inhibitor (TKI) therapies.^2^ The adverse effects of nilotinib are typically categorized into two main groups: hematological and nonhematological. Myelosuppression stands as the most prevalent hematological toxicity associated with nilotinib. Nonhematological toxicities include various disorders affecting the skin and subcutaneous tissues, gastrointestinal tract, fluid balance, musculoskeletal system, liver, kidneys, and general bodily functions. Notably, cardiovascular complications and elevated blood glucose levels occur more frequently in patients undergoing nilotinib treatment. These particular adverse events pose significant concerns, potentially limiting the long-term viability of nilotinib therapy and posing a risk to patient health and well-being.^13^ Given the adverse effects stemming from prolonged nilotinib use, it becomes crucial to accurately measure the levels of this medication in both biological samples and drug formulations. This precision is essential for ensuring the safety of treatment and enhancing patient survival rates.^14^

The review of the existing literature uncovered several methods for determining Nilotinib, such as high-performance liquid chromatography (HPLC),^15−20^ ultraperformance liquid chromatography–mass/mass spectrometry (UPLC–MS/MS),^21−23^ spectroscopy,^24^ spectrofluorometry,^11^ and voltammetry,^25,26^ Although possessing high sensitivity and selectivity, chromatography instruments are expensive, not widely accessible in many laboratories, and require intricate operation. Additionally, one of its drawbacks includes lengthy sample pretreatment procedures and the utilization of organic solvents, which pose risks to both the environment and human health.^14,15^ Electrochemical methods stand out among these techniques due to their cost-effectiveness, high sensitivity, rapid response times, and user-friendly nature,^27^ Electrochemical methods have shown high sensitivity in detecting organic molecules, including drugs and related compounds, in pharmaceutical products and biological fluids and their oxidizable property.^28−30^ The effectiveness of electrochemical sensors can be significantly enhanced by modifying their surface with nanostructures.^31−33^ Nanostructures, due to their extensive surface area and capacity to expedite electron transmission, have the potential to enhance sensitivity and lower detection limits in sensing applications.^34,35^ They can also improve selectivity by facilitating specific interactions with the analyte.^36,37^ Additionally, nanostructures contribute to enhancing the responsiveness and stability of sensors.^38,39^

Transition metal sulfides (TMSs) are commonly utilized as electrode materials in energy storage systems because of their high theoretical capacity and cost-effectiveness.^40−42^ However, TMSs have some handicaps, such as limited electrical conductivity and high-volume expansion. These handicaps lead to poor rate capability and fast capacity fading, respectively, when TMSs are used as electrode materials in energy storage systems. Many different approaches have been implemented to address these challenges.^43−46^ The synthesis of TMSs with a low particle size is important in improving the electrochemical performance of TMSs as it offers more phase boundaries. The high surface energies of nanoparticle-sized TMSs can cause undesirable levels of aggregation, which is to minimize the utilization of TMSs as an active material.^47,48^ Furthermore, the process of aggregation impedes the efficient transportation of electrons and carriers of charge. Consequently, numerous studies have attempted to incorporate nanoparticle TMSs into various carbon matrices or synthesize TMSs inside a carbon matrix.^49−55^ The presence of a carbon matrix acts as a barrier to prevent the gathering together of particles and enables the active material to be used to its highest extent, while also facilitating rapid electrochemical reactions. The intrinsic characteristics of carbon nanocomposites can be effectively modified through chemical doping with heteroatoms. Heteroatom-doped carbon is frequently synthesized by using nitrogen, sulfur, and boron atoms. For biosensing purposes, nitrogen doping has the potential to greatly improve carbon’s electron conductivity, sensitivity, and biocompatibility. It is important to consider that the carbon substrate and interface might have a significant impact on the doping properties.^56−59^

**Figure 2:**
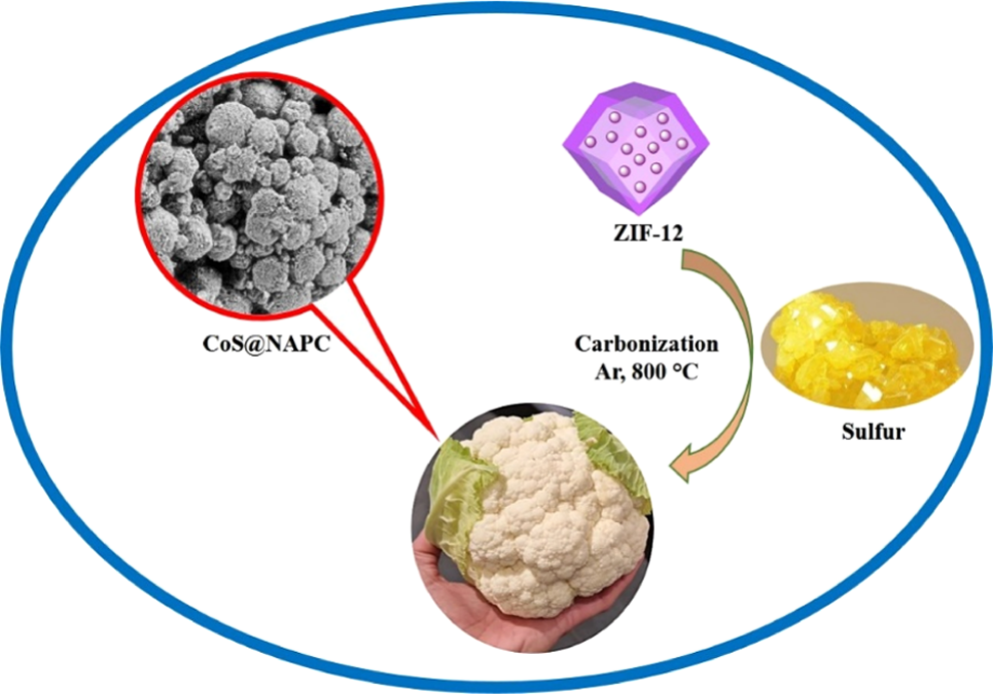
Illustration of the preparation path for the CoS@NAPC.

This work was inspired by the superior electrochemical performance of GCE electrodes modified with transition metal oxides/selenides embedded in a carbon matrix,^60−63^ and by the fact that MOFs are an excellent template for the synthesis TMS/carbon structures.^49,50,64−67^ As shown in Figure 2, after pyrolysis/sulfidation in one-pot, uniformly distributed nanosized CoS can be embedded in the in situ formed amorphous carbon matrix. As a result, CoS@NAPC composite structure was successfully synthesized and used as modifier material for GCE electrode on the detection of Nilotinib. The sensor’s performance and the electrochemical behavior of NLT were thoroughly investigated. Extensive validation studies were conducted, and the quantification of NLT in commercial samples of human urine and pharmaceutical preparations was achieved by using the developed CoS@NAPC/GCE.

## 2 Experimental Section

### 2.1 Materials Characterization

#### 2.1.1 Synthesis

Cobalt nitrate hexahydrate (0.5 g), benzimidazole (1.1 g), and NH_4_OH (0.5 mL) were dissolved in 100 mL of a methanol/toluene mixture (3:1) to form a homogeneous solution. The purple powder was collected by vigorously stirring for 3 h. After that, the suspension was centrifuged and dried at 70 °C for 12 h. Finally, ZIF-12 and sulfur powder (1:3 w/w) were mixed in a ceramic mortar. Then, the S/ZIF-12 powder was placed in the quartz boat and pyrolyzed at 800 °C for 2 h at a heating rate of 3 °C min^–1^ under an Ar atmosphere to fabricate CoS@NAPC.

#### 2.1.2 Materials

Cobalt nitrate hexahydrate (Co(NO_3_)_2_·6H_2_O), sulfur, and benzimidazole (C_7_H_6_N_2_) were supplied by Sigma-Aldrich. Methanol (CH_3_OH) and toluene (C_7_H_8_) were all provided by VWR Chemicals. None of the chemicals or reagents needed to be further refined, since they were all used immediately.

#### 2.1.3 Characterization Techniques

X-ray diffraction (XRD) patterns were collected on a Rigaku R int 2000 X-ray diffractometer between 2 and 80°/min with a scan rate of 2°/min to characterize the phase structure of ZIF-12 and CoS@NAPC. The morphologies and structure of materials were characterized by scanning electron microscopy (SEM) named Leo EVO-40 VPX, and transmission electron microscopy (TEM) images were attained with a Hitachi HT7700 instrument, functioning at 120 kV. The specific surface area of materials was determined from N_2_ adsorption–desorption measurements data obtained by using Micromeritics-TriStar 3000 based on Brunauer–Emmett–Teller (BET) multipoint method, and the corresponding pore size distribution was collected from the adsorption branches of the isotherms using Barret–Joyner–Halenda model. The vibrational modes of molecules were monitored by Raman spectroscopy (Raman microscope, RENISHAW INVIA, 532 nm excitation laser source). The X-ray photoelectron spectroscopy (XPS) measurement was carried out on a Thermo Scientific K-Alpha X-ray photoelectron spectrometer with an Al Kα monochromator source/1486.6 eV with a takeoff angle of 90° between the sample surface and the axis of the analyzer lens instrument in the range of 200–4000 eV spectrometer.

### 2.2 Electrochemical Measurements

#### 2.2.1 Materials and Chemical

In order to check additional specifics and detailed information, see the Supporting Information.

#### 2.2.2 Apparatus

Potentiostat/galvanostat device (PGSTAT128 N, Metrohm Autolab B.V., The Netherlands) fitted with a standard three-electrode system was used to carry out the electrochemical measurements. Measurements were made by using a glass electrochemical cell containing a 3 mm diameter working GCE, a platinum wire counter electrode, and an Ag/AgCl reference electrode immersed in 3 M KCl solution.

The pH of the prepared phosphate-buffered saline (PBS) solutions was measured with a HANNA pH meter (Edge Multiparameter pH Meter – HI2020).

#### 2.2.3 Preparation of the Working Electrode

Initially, the unmodified GCE was carefully cleaned according to a previously documented protocol.^68^ The GCE was polished using 0.05 mm alumina slurries. After polishing, the GCE was rinsed with ultradistilled water and cleaned with a 1:1 ethanol–water mixture for 5 min. The electrode was then rinsed with water and allowed to air-dry at room temperature for 10 min. To ensure thorough mixing of the composite, an ultrasonic laboratory bath was used. Then, 8.0 μL of the CoS@NAPC solution (0.5 mg/mL) was applied to the electrode surface. The modified electrode was allowed to dry naturally at room temperature. Once the solvent evaporated completely, the CoS@NAPC/GCE electrode was placed in an electrochemical cell for a series of tests.

#### 2.2.4 Real Samples Preparation

To prepare the standard solution from the capsule sample, 200 mg of powder from the contents of five NLT-labeled capsules were weighed and homogenized by grinding using a mortar and pestle. Subsequently, the powdered tablets were dissolved in a phosphate-buffered saline (PBS) solution at pH 2 to obtain a stock solution. The calibration data obtained earlier were utilized for quantifying the NLT content in the tablets through recovery experiments.^69^ Synthetic human urine was used as received. The NLT concentration in actual samples was assessed by using the standard addition technique.

## 3 Results and Discussion

### 3.1 Synthesis and Characterization of CoS@NAPC Material

**Figure 3:**
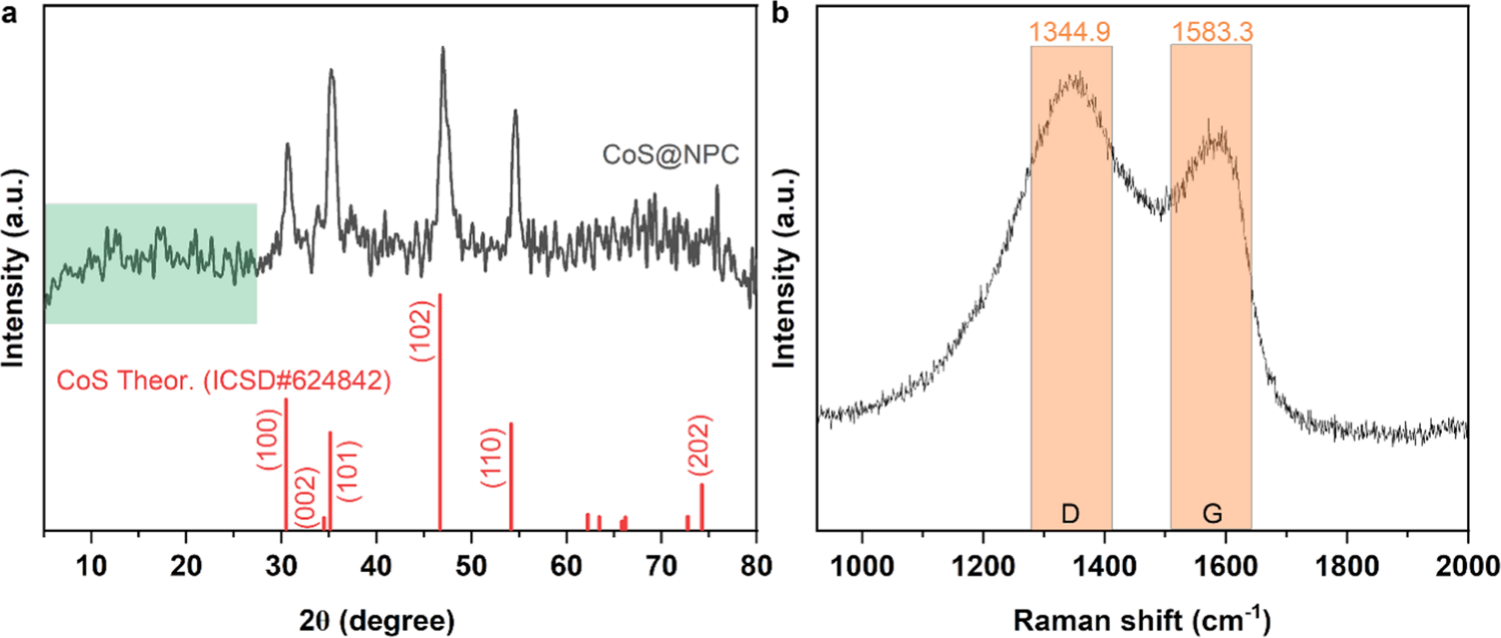
(a) XRD pattern and (b) Raman spectrum of the CoS@NAPC composite.

The SEM images of the ZIF-12 show a rhombic polyhedron structure with uniformly distributed diameters (∼1 μm or less, Figure S1c,d). The XRD patterns in Figure S1a and FT-IR analysis in Figure S1b demonstrate the successful synthesis of ZIF-12 and XRD patterns of ZIF-12 are well matched with the literature.^70^ After the pyrolysis process, the XRD method was utilized to examine the crystalline structure of the composite obtained. Figure 3 presents the XRD patterns of the CoS@NAPC material. It is evident that all reflections align with the simulated pattern derived from the single-crystal data of hexagonal CoS (ICDC#624842), confirming the highly crystalline nature of metallic cobalt within the structure. These reflections correspond well with the (100), (002), (101), (102), (110), and (202) planes at 2θ angles of 30.47, 34.50, 35.13, 46.69, 54.16, and 74.25°, respectively. Additionally, the presence of a small broad peak around 2θ = 20° confirms the existence of graphitic carbon, suggesting the amorphous nature of CoS@NAPC.^71,72^

The Williamson–Hall method was performed in order to calculate the average crystallite size (*D*) according to eq 1. Experimental data and fitted data are in best agreement with *R*^2^ = 0.96 (*D* ≈ 8 nm).
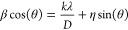
1The Raman spectra of CoS@NAPCs are depicted in Figure 3b, showcasing distinct D and G bands at 1344.85 and 1583.33 cm^–1^, respectively, indicative of the composite materials. These bands represent the graphitic and disordered phases within the structure, with the ratio of *I*_D_/*I*_G_ serving as a metric for graphitization extent.^73,74^ Notably, as the *I*_D_/*I*_G_ ratio declines, indicating enhanced graphitization, the overall structure’s graphitic nature becomes more pronounced. Conversely, when the *I*_D_/*I*_G_ ratio exceeds 1, it signifies a prevalence of defects within the samples. For the CoS@NAPCs, the calculated *I*_D_/*I*_G_ ratio stands at 1.17. The introduction of nitrogen atoms into the lattice structure, coupled with structural alterations, elevates the D-band intensity, corroborating an increase in structural defects and the emergence of an amorphous carbon structure.

**Figure 4:**
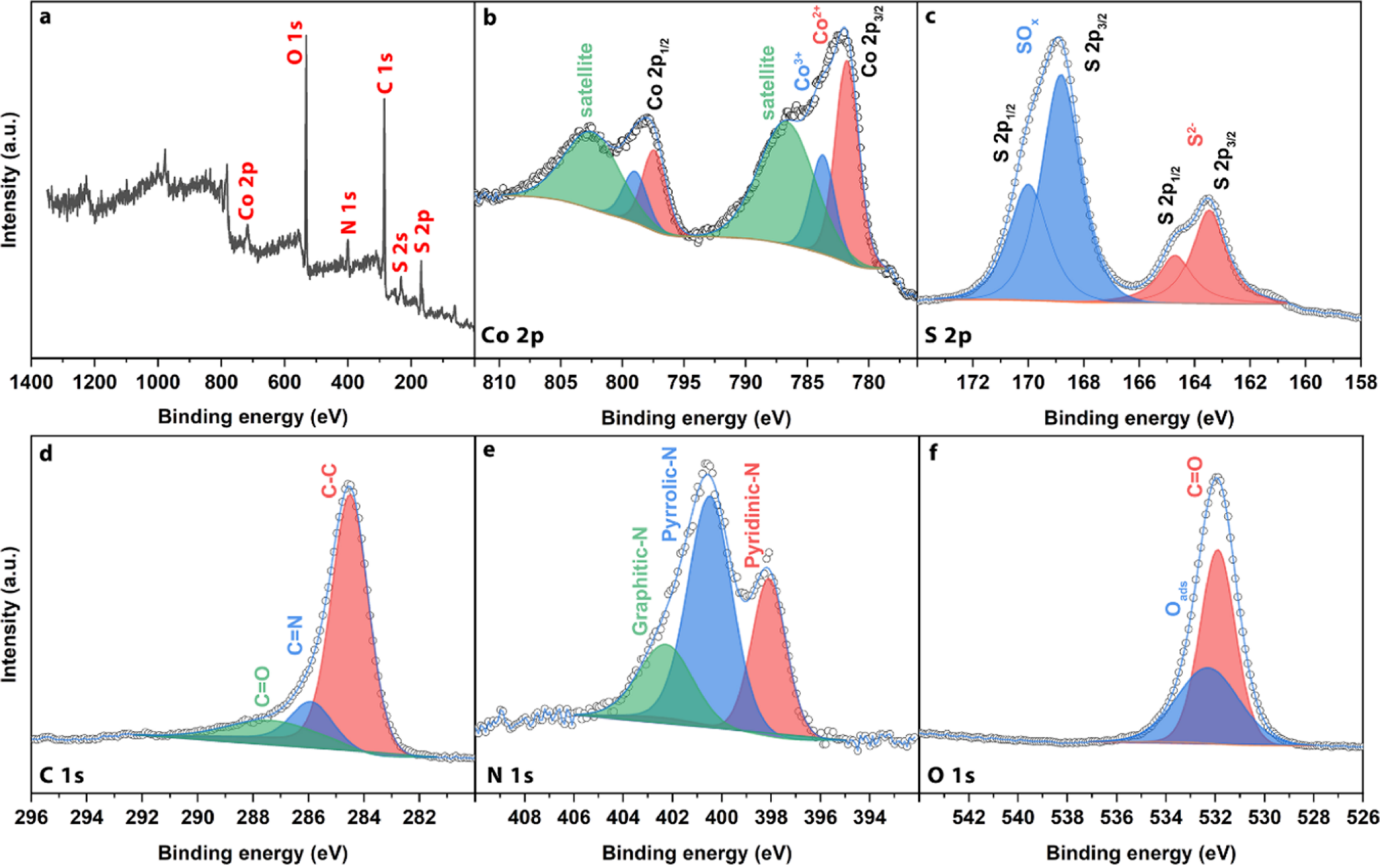
(a) XPS full spectrum and high-resolution XPS spectra of CoS@NAPC; (b) Co 2p, (c) S 2p, (d) C 1s, (e) N 1s, and (f) O 1s.

An XPS analysis was performed to enhance our comprehension of the surface composition and chemical states of the CoS@NAPC. Figure 4 illustrates both the survey and high-resolution XPS spectra of the Co, S, C, N, and O elements within the CoS@NPC composite. In Figure 4a, the XPS survey spectra reveal the presence of Co, S, C, N, and O atoms within the CoS@NPC samples. Examining the Co 2p spectrum of CoS@NAPC in Figure 4b, two doublets are evident, accompanied by a satellite doublet at 786.78 and 802.79 eV. The initial doublet, situated at 781.86 and 797.47 eV, corresponds to Co^2+^ within Co–S bonds, while the subsequent doublet, at 783.83 and 799.09 eV, is indicative of Co^3+^. The appearance of Co^3+^ may stem from the surface oxidation of Co or from the strong interaction between cobalt and the nitrogen-doped carbon matrix, potentially altering the electronic environment of Co.^75^Figure 4c shows the S 2p spectrum, where the two doublets can be identified as two types of sulfur in the composite. The first doublet at 163.49 and 164.72 eV are attributed to the Co–S bond indicating the formation of CoS. The second doublet at 168.83 and 170.02 eV can be assigned to the C-bonded SO_*x*_.^76^ In Figure 4d, the high-resolution spectrum of C 1s is depicted, revealing three discernible peaks at approximately 284.50, 285.92, and 287.57 eV. These peaks correspond to sp^2^ hybridized C–C, C–N, and C–O bonds, respectively, when the spectrum is deconvoluted. Figure 4e illustrates the presence of nitrogen-doped graphitic carbon, as made evident by the N 1s spectrum. Moreover, the N 1s spectrum reveals three distinct peaks, namely, pyridinic-N (398.13 eV), pyrrolic-N as Co–N_*x*_ (400.49 eV), and graphitic-N (402.34 eV).^77,78^ Lastly, the O 1s spectrum depicted in Figure 4f provides evidence of the presence of C–O bonds and adsorbed oxygen groups on the surface.

**Figure 5:**
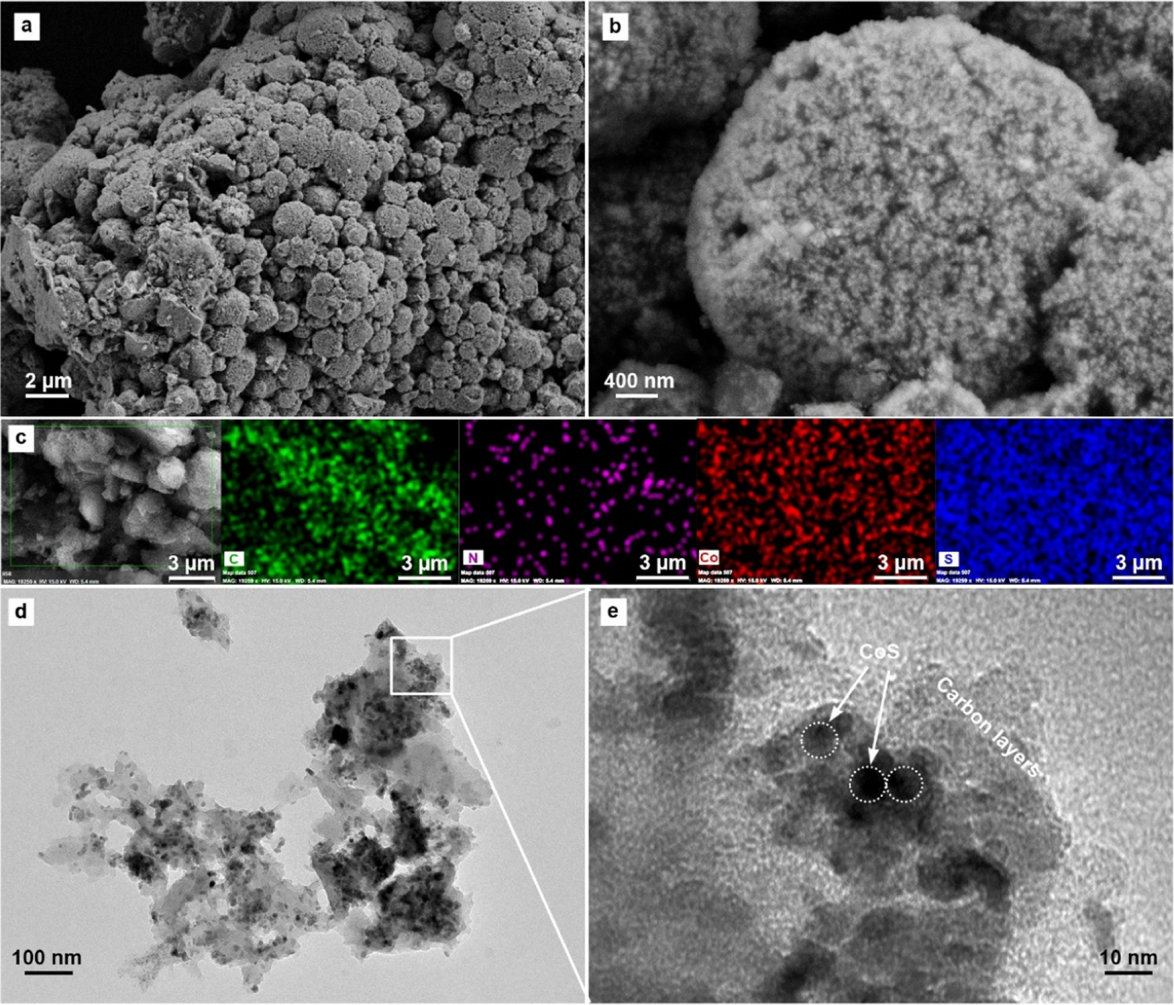
Morphological and microstructural characterizations. (a, b) SEM images of CoS@NAPC composite, (c) SEM image and the corresponding EDS mappings of CoS@NAPC composite, and (d, e) TEM images of CoS@NAPC composite.

SEM images in Figure 5a,b offer a revealing glimpse into the morphology of the CoS@NAPC composite. Unlike the rhombic polyhedron characterizing ZIF-12, as previously detailed in our study,^50^ these images depict porous cauliflower-like structures, each spanning a few micrometers. This distinctive morphology emerges from the interplay of sulfidation and pyrolysis during synthesis, wherein the decomposition of benzimidazole ligands triggers shrinkage, ultimately giving rise to the observed porous architecture. Figure 5c illustrates the EDS mapping of CoS@NAPC, vividly revealing the distribution of the Co, S, N, and C elements across the surface. The atomic compositions were as follows: C (31.40%), N (21.45%), Co (11.83%), S (17.04%), and O (18.27%). TEM analysis was conducted on the composite particle with results depicted in Figure 5d,e. These images unequivocally reveal the embedding of CoS nanoparticles onto the surface of the amorphous carbon layers. In Figure 5e, the TEM image highlights the CoS nanoparticles, which exhibit a relatively uniform size distribution with diameters ranging from approximately 5–10 nm. This is consistent with XRD analysis. The combination of XRD, SEM/EDS, and TEM findings conclusively verifies the effective synthesis of the CoS@NAPC compound.

**Figure 6:**
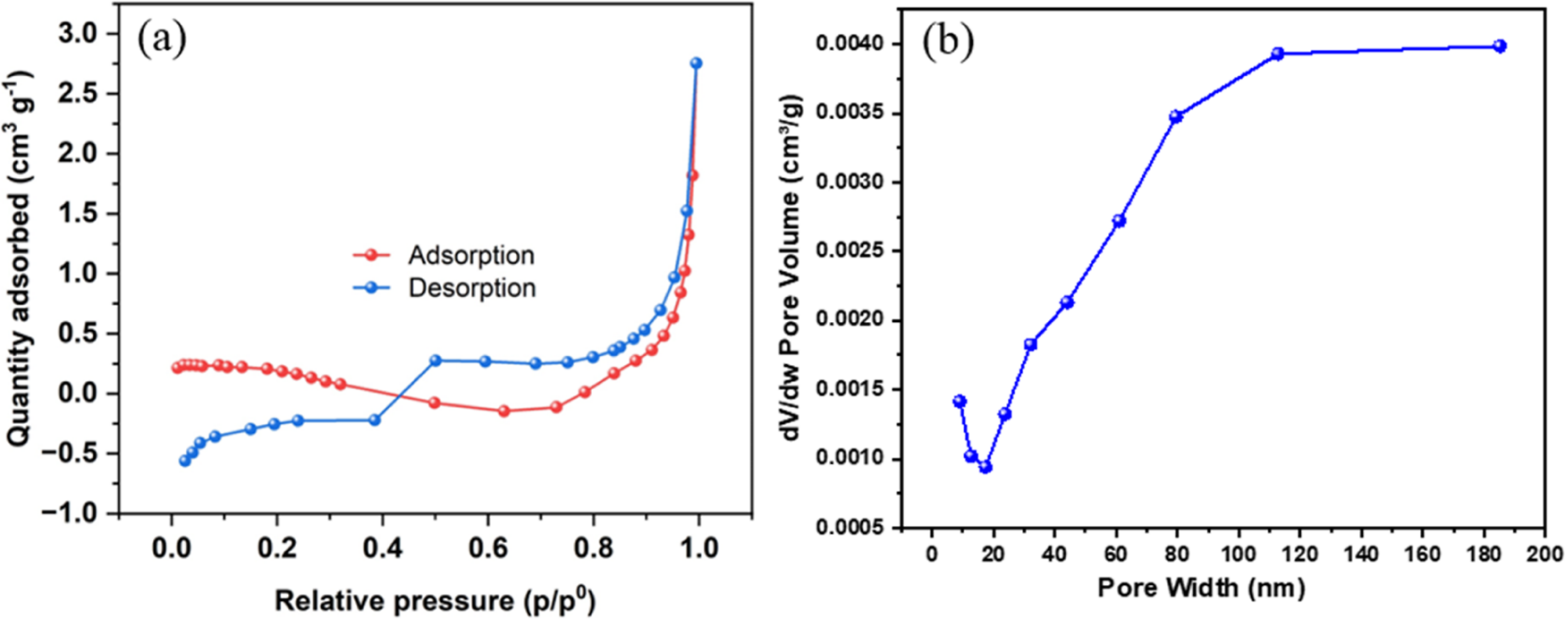
N_2_ adsorption–desorption isotherms (a) and pore size distribution curve (b) of CoS@NAPC composite.

As illustrated in Figure 6, the BET surface analysis revealed a surface area of 0.255 m^2^/g for the CoS@NAPC composite structure, and the hysteresis loop in its N_2_ adsorption/desorption curve is similar to the type of IV isotherm. The low surface area of the CoS@NAPC material may be due to the fact that the pores are completely filled with elemental sulfur during the sulfurization process. In addition, the pore structure of CoS@NAPC material was studied by pore size distribution curve. As shown in Figure 6b, the pore volume of CoS@NAPCis 0.000887 cm^3^ g^–1^. The fact that the pore size value is so low supports the idea that it may be due to the filling of the pores with elemental sulfur.

### 3.2 Electrochemical Characterization of Nilotinib

Differential pulse voltammetry (DPV) measurements were performed in a 0.1 M PBS solution (pH 2) by using a 50 mV pulse amplitude, 0.01 s pulse time, 5 mV potential step, and a 50 mV/s scan rate to scan from +0.7 to +1.5 V. As seen in Figure S3, NLT’s anodic peak current on the modified electrode (14.20 μA, 1.118 V) was higher than its value on the bare electrode (7.33 μA, 1.1331 V). This observation implies the oxidation of NLT at the electrode interface, with the modified electrode yielding a response approximately twice as potent, indicative of heightened sensitivity. Such behavior is attributed to the enhanced conductivity and augmented surface area activity conferred by the modified electrode.

Cyclic voltammetry (CV) was performed in a 0.1 M KCl electrolyte solution using 5.0 mM [Fe(CN)]_6_^3–/4–^ as the model analyte. The potential was scanned from −0.5 to +1.0 V at a scan rate of 50.0 mV/s. Two peaks were observed: an anodic peak at −0.0361 and +0.1518 V, and a cathodic peak at +0.3129 and +0.2397 V for the bare electrode and modified electrode, respectively (Figure S4a). The cathodic and anodic peak currents showed significant increases. The significant decrease in peak potential separations (Δ*E*_p_) observed for CoS@NAPC/GCE suggests a developed electron transfer rate and a greater efficient surface area of the designed electrode. The potential difference between the two peaks is greater than 0.059 V, indicating that the electrochemical behavior of NLT is irreversible.^79^

The sensitivity of the electrochemical sensor hinges primarily on the electroactive surface area (ESA), governing the extent of the analyte interaction with the electrode surface. Hence, both the developed and bare electrodes’ ESA is computed using the Randles–Sevcik equation (eq S1). Figure S5 shows the relationship between the peak current and the square root of the scan rate, as previously reported.^80^ As a result, the surface area of the bare GCE was found to be 0.0911 cm^2^, while the CoS@NAPC/GCE had a surface area of 0.1869 cm^2^. These results indicate that CoS@NAPC/GCE has an excellent electron transfer capability and high conductivity. This finding supports our hypothesis that modification increases the surface area of the electrode, which is advantageous for enhancement.

Electrochemical impedance spectroscopy (EIS) measurements were conducted in a 0.1 M KCl solution using 5.0 mM [Fe(CN)]_6_^3–/4–^ as the model analyte. The frequency ranged from 10^5^ to 10^–1^ Hz at an open-circuit potential of +0.1 V, with a voltage amplitude of 10 mV. The variation in charge transfer resistance (*R*_ct_) is represented by the semicircle’s diameter in the Nyquist diagram, which reflects changes at the electrode surface. Additionally, the capacitance helps to assess the electrical characteristics of the electrode. The *R*_ct_ values and capacitance were calculated using NOVA 2.1.6 software.

EIS proves to be a valuable method for thoroughly examining the conductivity properties of electrode surfaces. Analysis of Nyquist plots (Figure S4b) generated via EIS revealed *R*_ct_ of 6074.0 Ω for the bare GCE and 2938.9 Ω for CoS@NAPC/GCE. The significant reduction observed in the EIS curves can be based on the excellent electrical conductivity of CoS@NAPC. Furthermore, the electrical properties of an electrode can be evaluated by determining capacitance. The double-layer capacitance (CDL) values, represented as constant phase element (CPE), derived from EIS analysis were 0.6452 μF for the unmodified electrode and 1.5593 μF for CoS@NAPC/GCE. This substantial disparity underscores the discernible enhancement in capacitance resulting from the modified electrode’s advanced structural composition and electrochemical characteristics.

The electrochemical responses of 0.1 mM NLT were measured using both an unmodified GCE and a CoS@NAPC/GCE. Electrochemical parameters determined by DPV, CV, and EIS measurements are listed in Table S1.

### 3.3 Optimizing Electrode Modification

The sensitivity of CoS@NAPC/GCE was optimized through thorough analysis and modification of multiple critical parameters. These included the concentration and volume of the nanocomposite, the type and pH of the supporting electrolyte, and the scan rate. The following subsections delineate the process of optimization.

#### 3.3.1 Choosing of Supporting Electrolyte

The energy consumption and conductivity of the solution are greatly influenced by the supporting electrolyte selection. DPV with 0.1 mM NLT was used to test a variety of electrolytes including phosphate buffer saline (PBS), Britton–Robinson (BR), KCl, acetate buffer (AC), and HCl. PBS provided the best response in terms of peak current and shape and was therefore selected for the entire experiment (Figure S6a).

#### 3.3.2 The Impact of Nanocomposite Concentration

The electrode surface was explored within the range of 0.1–2.0 mg/mL. The peak current reached its maximum at 0.5 mg/mL. However, higher concentrations resulted in challenges such as aggregation or overlapping of binding sites, thereby diminishing the effective adsorbent surface area (Figure S6b).

#### 3.3.3 Nanocomposite Volume

It was investigated how the nanocomposite volume affected the electrode surface between 2.0 and 10.0 μL. The best result was observed with 8.0 μL. Volumes exceeding this caused the modifier layer to adhere less to the surface and inhibit analyte diffusion, resulting in a notable decrease in the sensitivity of CoS@NAPC/GCE. Therefore, 8.0 μL was determined as the optimal compound volume (Figure S6c).

#### 3.3.4 The Impact of pH

The evaluation of pH-buffered PBS has been investigated as a major factor in the performance of the electrochemical sensor. The effect of pH on the efficiency and capacity of NLT was studied within a pH range of 1.0–7.0 (Figure S7a). The highest peak currents were recorded at pH 2.0, which was thus determined to be the optimal condition for subsequent experiments. Figure S7b also shows that the oxidation peak potential of NLT shifts negatively as the pH increases from 1.0 to 7.0, suggesting that protons are involved in NLT oxidation.

#### 3.3.5 The Impact of Scan Rate

It was examined how the scan rate affected the peak currents (*I*_pa_) and potentials (*E*_pa_) of NLT on the improved electrode surface. To explore the reaction mechanism, it was recorded cyclic voltammograms (CVs) of NLT across a scan rate range of 10.0–225.0 mV/s (Figure S8a). As shown in Figure S8b, the oxidation peak current increased with the square root of the scan rate within this range. As scan speeds increase, so do the NLT background current and the peak shape distortion. A lower oxidation current toward the electrode results from the diffusion layer moving away from the electrode when the scan rate is too slow, which lowers the oxidation peak’s current.^81^ Consequently, for the experiments that followed, a scan rate of 50 mV/s was determined to be optimal. The equation *I*_pa_ (μA) = 0.122υ^1/2^ – 0.443 (*R*^2^ = 0.996) suggests that at the sensor surface, NLT’s electron transfer reaction occurs in a diffusion-controlled manner.^82^ The number of electrons involved in the electrochemical oxidation of NLT on CoS@NAPC/GCE was determined using eq S2, which is applicable for irreversible reactions.^83^ The estimation yielded a transfer of approximately 2.2 (∼2e^–^) electrons during the NLT electro-oxidation process. Based on the results obtained for NLT on CoS@NAPC/GCE, the possible electro-oxidation mechanism for NLT is proposed in Scheme S1.^84^

After the optimization process was completed, experimental parameters were established. These included a PBS-supplemented electrolyte at pH 2.0, a scan rate of 50 mV/s, a composite concentration of 0.5 mg/mL, and a composite volume of 8.0 μL.

### 3.4 Analytical Performance of the Sensor Created for the Detection of Nilotinib

**Figure 7:**
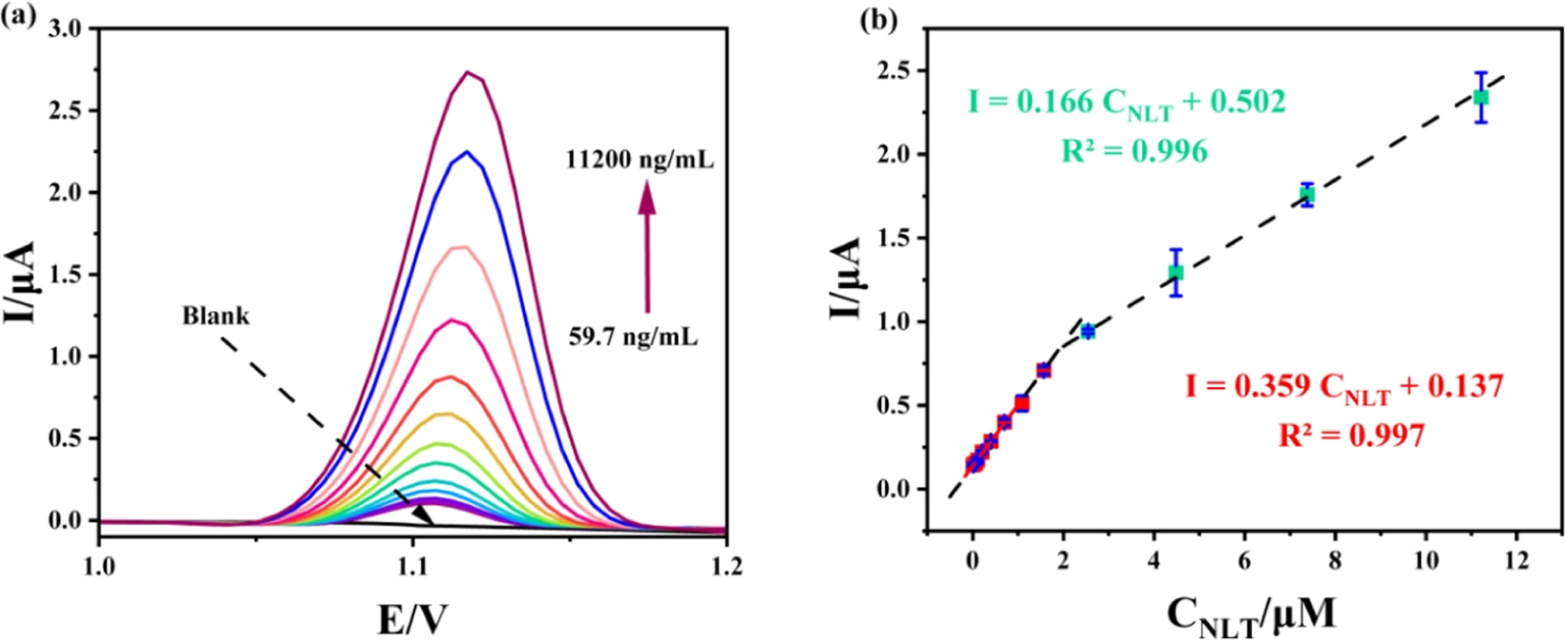
(a) DPV curves obtained by increasing concentrations of NLT at CoS@NAPC/GCE in 0.1 M PBS pH 2.0 (59.7–11,200 ng/mL). (b) Plot of peak current vs concentration of NLT.

The quantitative performance of the fabricated electrode for NLT analysis was investigated using DPV under the optimized conditions at the CoS@NAPC/GCE surface. As shown in Figure 7a, the anodic current of NLT increases linearly with concentration within the ranges of 59.7–1570 and 1570–11200 ng/mL. This relationship is described by the following linear regression equations: *I* = 0.359*C*_NLT_ + 0.137 (*R*^2^ = 0.997) and 0.166*C*_NLT_ + 0.502 (*R*^2^ = 0.996) (Figure 7b). Kinetic limitations at higher concentrations are probably the cause of the reduction in sensitivity (slope) in the second linear range.^85^ Limits of detection (LOD) and limits of quantification (LOQ) were carefully determined using the equations LOD = 3σ/*m* and LOQ = 10σ/*m*, respectively.^86^ Thanks to these calculations, the least detectable and measurable concentrations of the analyte can be comprehensively evaluated. In our analysis, LOD and LOQ values were established as 11.8 and 39.3 ng/mL, respectively.

Table 1 presents various characteristic parameters, including the detection method, linear concentration range, and detection limit, from other reported methods. Notably, the detection limit achieved with the method discussed here is substantially lower than those obtained with most other methods. Moreover, the sensor offers a good linear range. These findings suggest that the proposed CoS@NAPC/GCE could serve as a promising alternative for NLT detection.

**Table 1:** Comparison of the Effectiveness of Various Techniques for NLT Detection

method	linear range (ng/mL)	LOD (ng/mL)	ref
HPLC-UV	125–7000	90.0	(87)
spectroscopy	1000–9000	225.0	(24)
spectroscopy	40–700	8.0	(24)
LC-MS/MS	6–1500		(21)
RP-HPLC	100–600	7.0	(15)
spectrofluorometry	100–1000	25.0	(11)
selective extraction	10^4^–10^7^	2000.0	(14)
SWV (GCE (the presence of SLS))	20–2000	6.33	(26)
DPV (ds-DNA/In^3+^/NiO RLHNSs/CPE)	10–50,000	0.62	(25)
DPV (CoS@NAPC/GCE)	59.7–1570 and 1570–11,200	11.8	this work

### 3.5 Study of Repeatability, Reproducibility, Potential Interferents, and Stability

Ten consecutive cycles were obtained, and the relative standard deviation (%RSD) was computed to evaluate the repeatability of the developed CoS@NAPC/GCE (Figure S9a). The repeatability was found to be perfect with an RSD percentage of 3.29% for these 10 cycles.

Furthermore, 8 electrodes were created under the same circumstances in order to assess the CoS@NAPC/GCE’s reproducibility. For each electrode, DPV signals were obtained (Figure S9b). With a low percentage RSD of 2.55%, the current variations demonstrated flawless reproducibility.

In addition to being repeatable and reproducible, the sensor needs to show selectivity for the intended analyte. Several coexisting interference materials that are frequently present in actual samples were added to the electrochemical cell in order to evaluate selectivity. Using a CoS@NAPC/GCE sensor, the interference effects of KCl, dopamine (DOPA), ascorbic acid (AA), l-arginine (l-A), uric acid (UA), KNO_3_, Na_2_SO_4_, and d-glucose were investigated (Figure S9c). NLT was mixed with each component in ratios of 1:100 or 1:1000 to calculate the RSD values, illustrating the impact of interference. The RSD values obtained were ≤3.75% (Table S2). These results indicate that the interference materials did not affect the performance of the CoS@NAPC/GCE sensor in the analysis.

For stability testing, the electrode was stored at room temperature in the laboratory, and measurements were repeated over 5 days (Figure S9d). After 5 days, the electrode retained 95.45% of its initial response. These results indicate that CoS@NAPC/GCE has good stability and can be used to detect NLT.

### 3.6 Application of the CoS@NAPC/GCE Sensor

The ability of the recently created sensor to identify NLT in a range of real samples was assessed, such as capsules and human urine, in order to determine its practical utility. The studies were carried out in triplicate in order to calculate the standard deviations and recoveries. The oxidation peaks in the DPV diagrams were analyzed, and the standard addition technique was used to compute the recoveries and RSDs, which fall into the ranges of 98.41–102.03 and 0.57–2.63%, respectively (see Table 2). These findings suggest that the technique, which uses the CoS@NAPC/GCE-modified electrode, is a good fit for precisely detecting NLT in actual samples, exhibiting high precision and accuracy.

**Table 2:** Analysis of NLT in Real Samples by DPV and Recovery Studies

specimen	added (μM)	found (μM)^a^	RSD (%)	recovery (%)
capsules	0.3	0.306	2.63	102.03
	0.4	0.396	1.11	99.06
	0.5	0.507	0.90	101.48
human urine	0.3	0.299	0.57	99.75
	0.4	0.393	1.93	98.41
	0.5	0.502	1.74	100.51

a Three duplicate measurements were used to calculate the mean value.

## 4 Conclusions

Ultimately, CoS nanoparticles evenly distributed within an amorphous carbon network were created, analyzed, and tested as a modifier for a glassy carbon electrode in detecting Nilotinib. The electrochemical methods were optimized, validated, and applied. The developed sensor successfully determined Nilotinib in human urine samples and pharmaceutical dosage forms with satisfactory results. Consequently, it can estimate Nilotinib with acceptable percentage recoveries and low %RSD values. The sensor demonstrated good selectivity and a high recovery. The suggested methods offer advantages, such as simplicity and time efficiency. Additionally, these methods are well suited for quality control due to their straightforwardness and low environmental impact.

## Supporting Information Available

XRD pattern, FT-IR, and SEM images of the ZIF-12, SEM and TEM images of CoS@NAPC, DPV results, CVs and EIS results, plausible oxidation mechanism of NLT, etc. (PDF)
