# Supplementary material for: Novel Electrochemical Approaches for Anticancer Drug Monitoring: Application of CoS@Nitrogen-Doped Amorphous Porous Carbon Composite in Nilotinib Detection
Source: ACS Omega. 2024 Dec 31;10(1):261–71. doi: 10.1021/acsomega.4c05505 (PMC11740382; doi:10.1021/acsomega.4c05505)
Supplement: Supplementary file 1 — ao4c05505_si_001.pdf [file ao4c05505_si_001.pdf]

Supporting Info

Novel Electrochemical Approaches for Anticancer Drug Monitoring: Application of CoS@nitrogen-doped amorphous porous carbon Nano composites in Nilotinib Detection

Merve Yıldır[1,2,*], Asena Ayse Genc[1,2], Nesrin Buğday[3], Nevin Erk[1,*] ,Naeimeh Sadat Peighambardoust[4], Umut Aydemir[4], Sedat Yaşar[3,*]

[1] Ankara University, Faculty of Pharmacy, Department of Analytical Chemistry, 06560, Ankara, Turkey

[2]Ankara University, Graduate School of Health Sciences, 06110, Ankara, Turkey

[3]İnönüÜniversity, Faculty of Science and Art, Department of Chemistry, 44280, Malatya, Turkey

[4]Koç University, Boron and Advanced Materials Research Center (KUBAM),34450, Istanbul, Turkey

asenaaysegenc@gmail.com, nbugday7@gmail.com, npeighambardoust@ku.edu.tr, uaydemir@ku.edu.tr,

*Corresponding authors: sedat.yasar@inonu.edu.tr, erk@pharmacy.ankara.edu.tr and ecz.merveyildir@gmail.com

Materials and chemical for Electrochemical Measurements

Nilotinib (Tasigna) waspurchasedfromNovartisPharmaceuticals. Potassiumhexacyanoferrate (III) ($K_3Fe(CN)_6$, 99.5 %), L-ascorbicacid (99.0 %), glucose (99.5 %), dopaminehydrochloride (99.0 %), uricacid (99.0 %), L-arginine (98.0%), L-cysteine (97.0%), potassiumchloride (KCl), potassiumnitrate (KNO_3), Sodiumsulfate (Na_2SO_4), acetaminophen, Sodiumchloride (NaCl)weresuppliedfromSigmaAldrichCo. (Germany). Ethanol (C_2H_5OH), Acetonitrile (CH_3CN), sodium hydroxide (NaOH) were supplied from Merck (Darmstadt, Germany). Benzimidazole (BIM), Cobaltacetatetetrahydrate ($(CH_3COO)_2Co.4H_2O$),andammoniumhydroxide (NH_3, 28–30% aqueoussolution) werepurchasedfrom Alfa Aesar.Allchemicalsareanalyticalreagentsandareused as receivedwithoutanyfurtherpurification.

Allsolutionpreparationprocesseswerecarriedoutusing Milliporewaterwith a resistivity of 18.2 MΩ. Allexperimentswereconducted at thesametemperature (~ 24 °C).

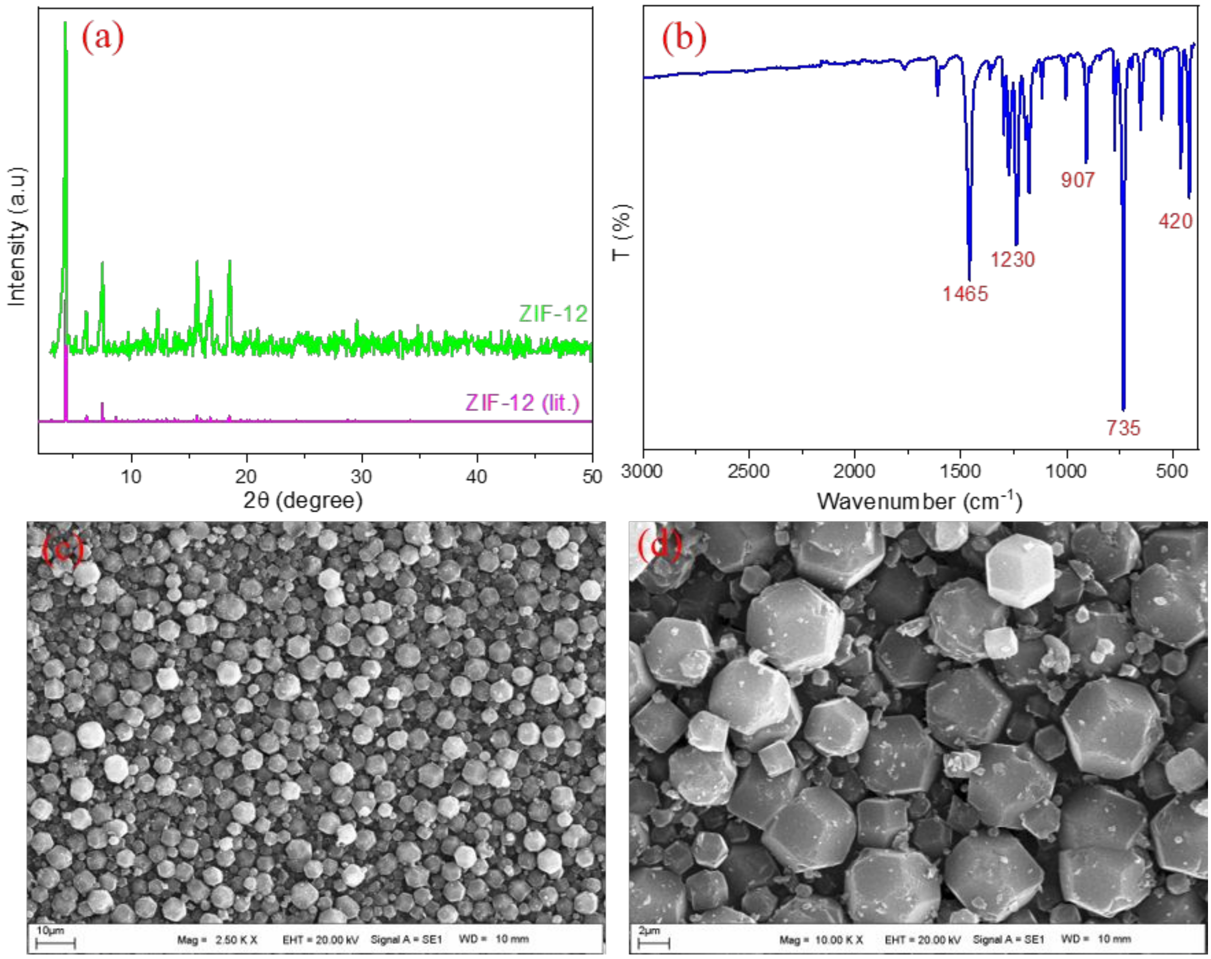

Figure S1. XRD pattern (a), FT-IR (b), and SEM images (c,d) of the ZIF-12 in different magnifications.

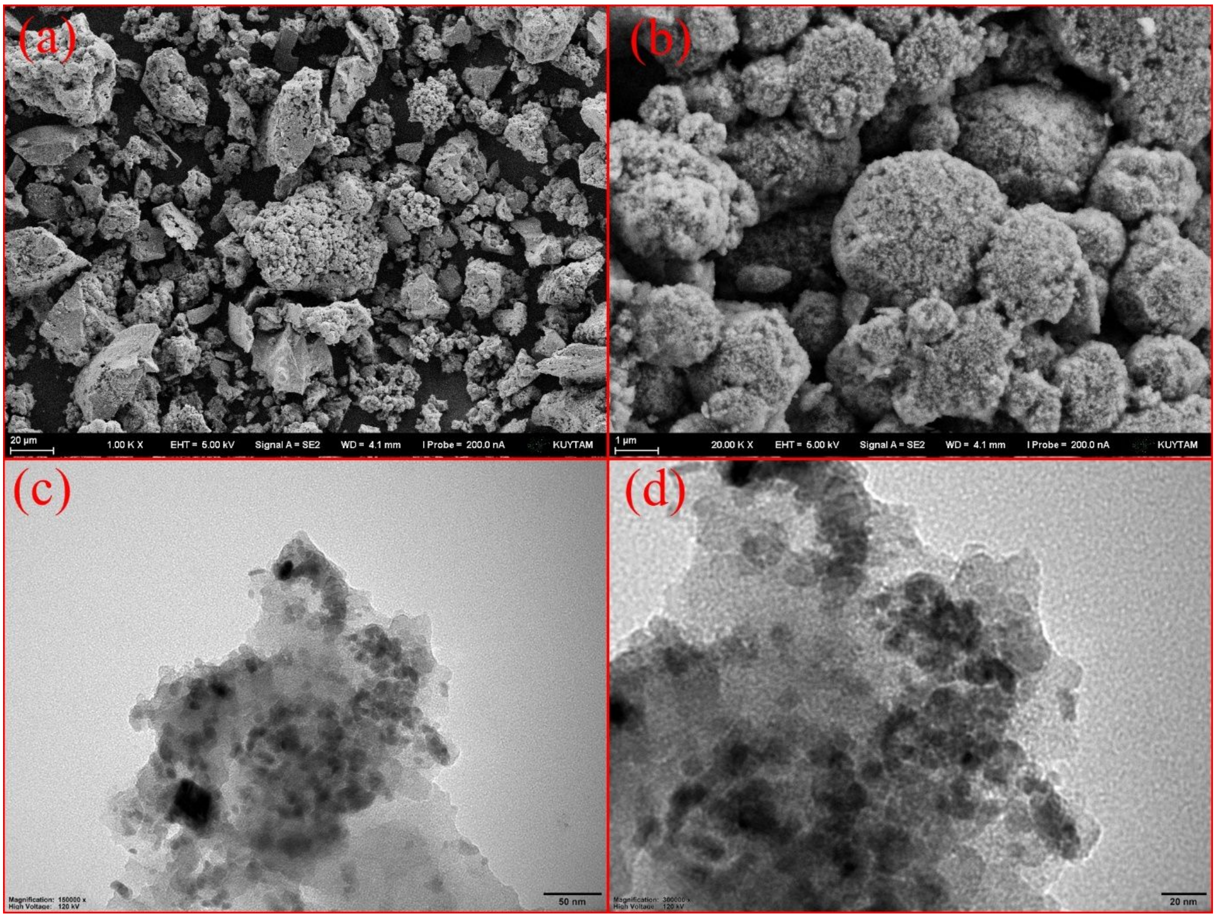

Figure S2. SEM (a,b) and TEM (c,d) images of the CoS@NAPC composite in differentmagnifications.

Calculation of the avarage crystallite size by Williamson-Hall method;

$$\beta \cos(\theta) = \frac{k\lambda}{D} + \eta \sin(\theta)$$

2θ	FWHM
30.7646	0.464052
35.3203	0.695035
47.0381	0.508033
54.7397	0.629559

Williamson-Hall method was performed in order to calculate the average crystallite size (D) and the best agreement between the experimental and fitted data with $R^2 = 0.96$ (D ≈ 8 nm).

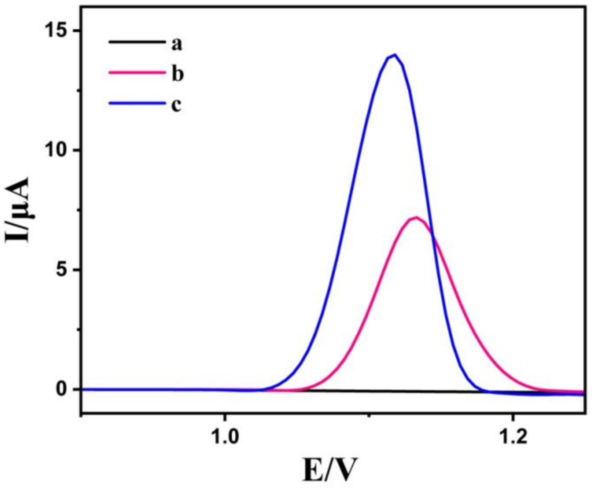

Figure S3. DPV results of 0.1 mM NLT in 0.1 M PBS buffer at pH 2.0, (—a) blank, (—b) bare GCE and (—c) CoS@NAPC/GCE.

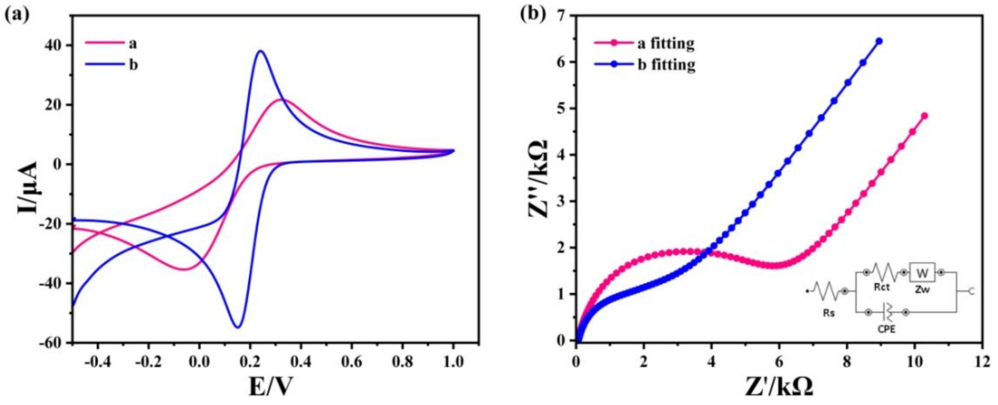

Figure S4. CVs(a) and EIS(b) (50.0 mV/s) in 0.1 M KCl and 5.0 mM $[Fe(CN)_6]^{3-/4-}$ solution of bare GCE (—a), CoS@NAPC/GCE (—b).

Table S1. Electrochemical parameters determined by DPV, CV and EIS measurements of GCE and CoS@NAPC/GCE.

Methods	Parameters	Electrode	
		GCE	CoS@NAPC/GCE
**DPV**	**Ip (μA)**	7.33	14.20
**CV**	**Δep (V)**	0.3491	0.0879
**CV**	**ESA (cm^2)**	0.0911	0.1869
**EIS**	**Rct (Ω)**	6074.0	2938.9
**EIS**	**Capacitance (μF)**	0.6452	1.5593

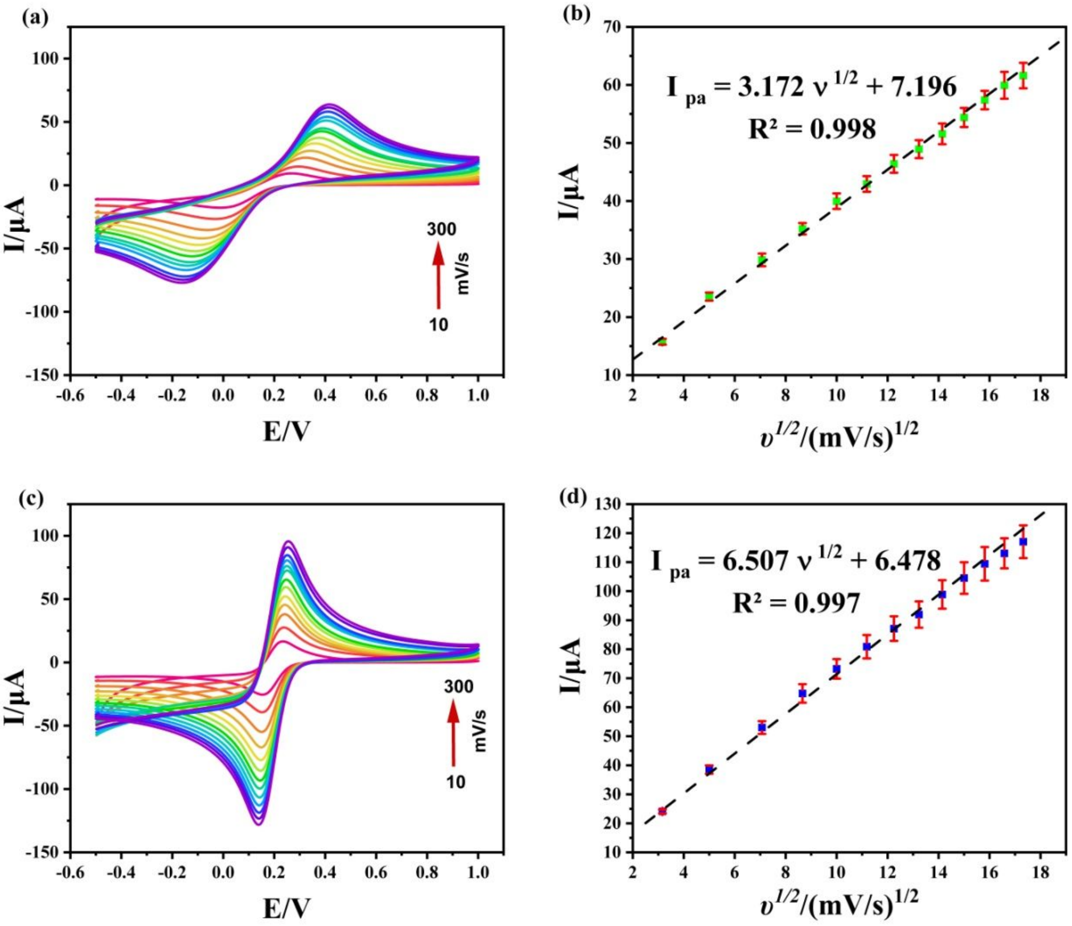

Figure S5. CV curves of 1.0 mM $[Fe(CN)_6]^{3-/4-}$ in 0.1 M KCl at varying scan rates (10 to 300 mV/s) for (a) bare GCE, (b) the plot of anodic peak current versus square scan rate; CV curves of 1.0 mM $[Fe(CN)_6]^{3-/4-}$ in 0.1 M KCl at varying scan rates (10 to 300 mV/s) for (c) CoS@NAPC/GCE, (d) the plot of anodic peak current versus square scan rate.

$$Ipa = 2.69 \times 10^5 n^{3/2} A D^{1/2} C v^{1/2} \quad (S1)$$

(It is expressed as Ipa: anodic peak current, n: number of electrons, A: electroactive surfacearea (cm^2), D: diffusion coefficient (cm^2/s), C: concentration (mol/cm^3), v: scan rate (mV/s^1).)

$\alpha n = 0.048/(Ep - Ep/2)$ $(S2)$(α:charge transfer coefficient$n$: number of electrons, $Ep/2$: potential at the half-peak current)

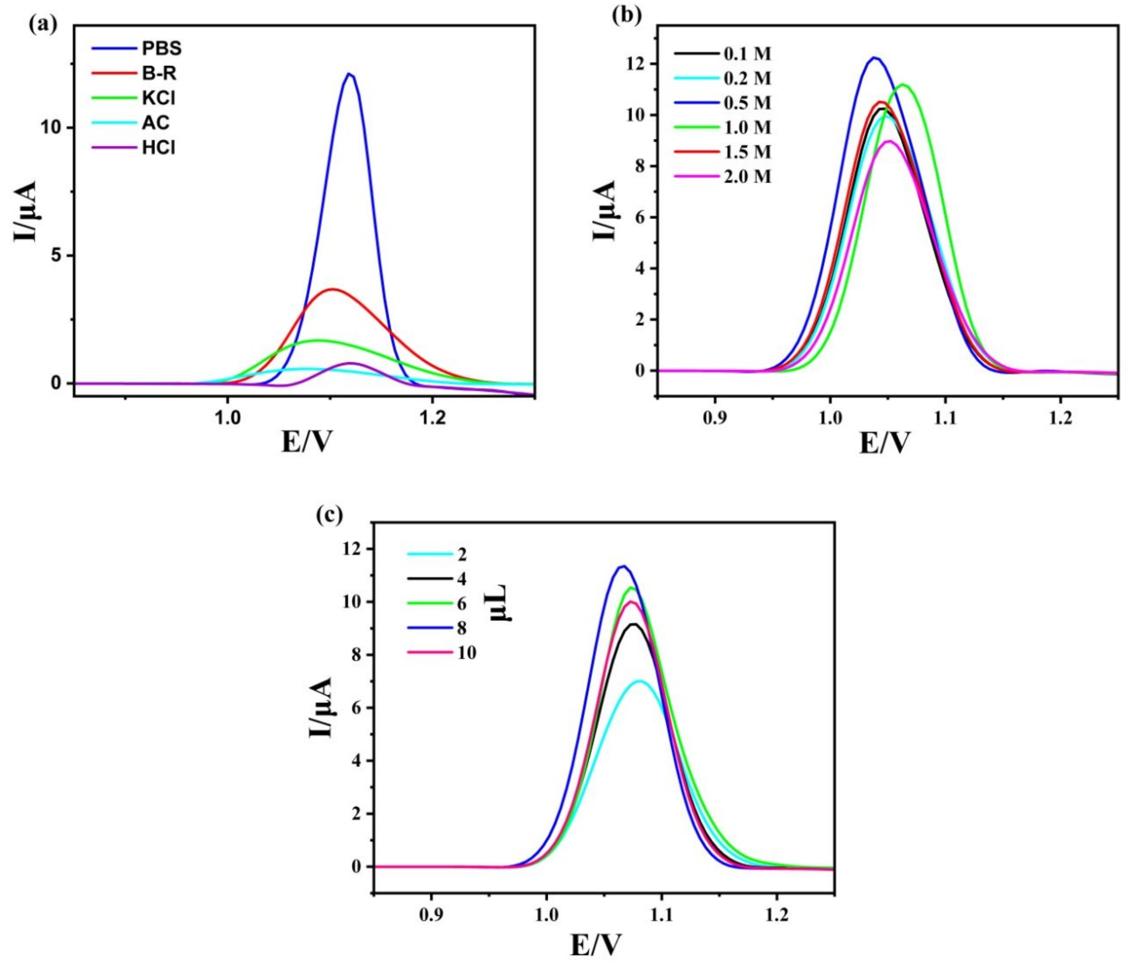

Figure S6. DPVs of different (a) electrolytes; (b) concentrations of the composite from 0.1 to 2.0mg/mL; and (c) amounts of composite.

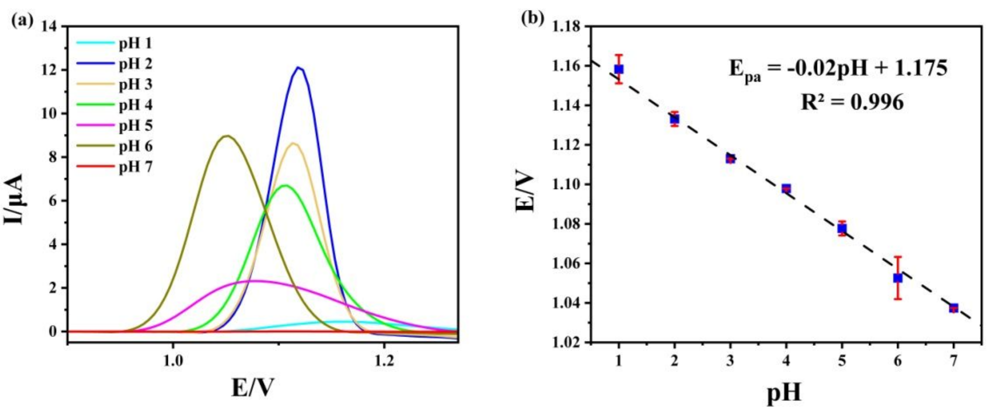

Figure S7. (a) DPV curves obtained from CoS@NAPC/GCE in 0.1 M PBS containing 0.1 Mm NLT at various pH values, and(b) the dependence of peak potential (Ep) on the pH value.

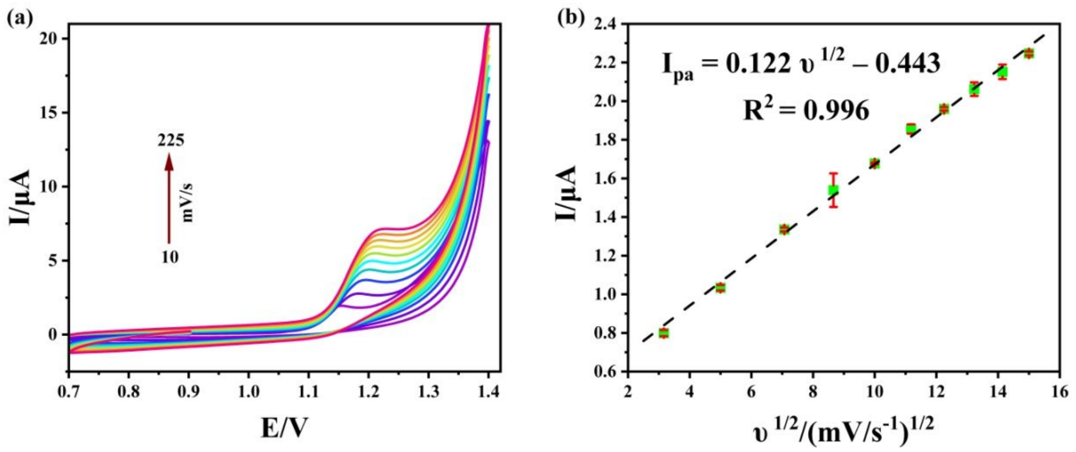

Figure S8. (a); CVs of 0.1 mM NLT according to scan rates (10.0–225.0 $mV.s^{-1}$), (b) on CoS@NAPC/GCE Dependence of peak current on the square root of scan rate.

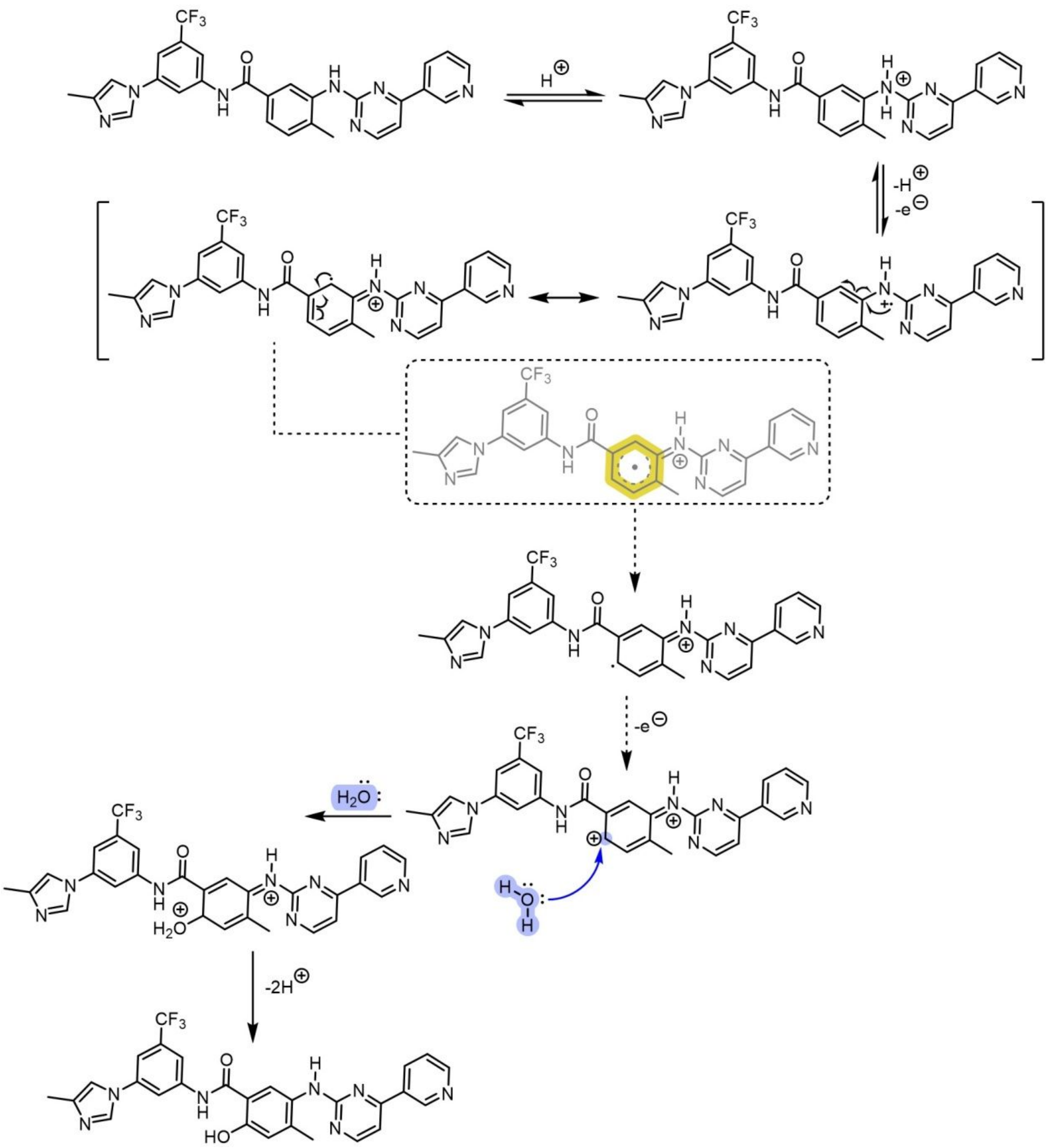

Scheme S1. The plausible oxidation mechanism of NLT.

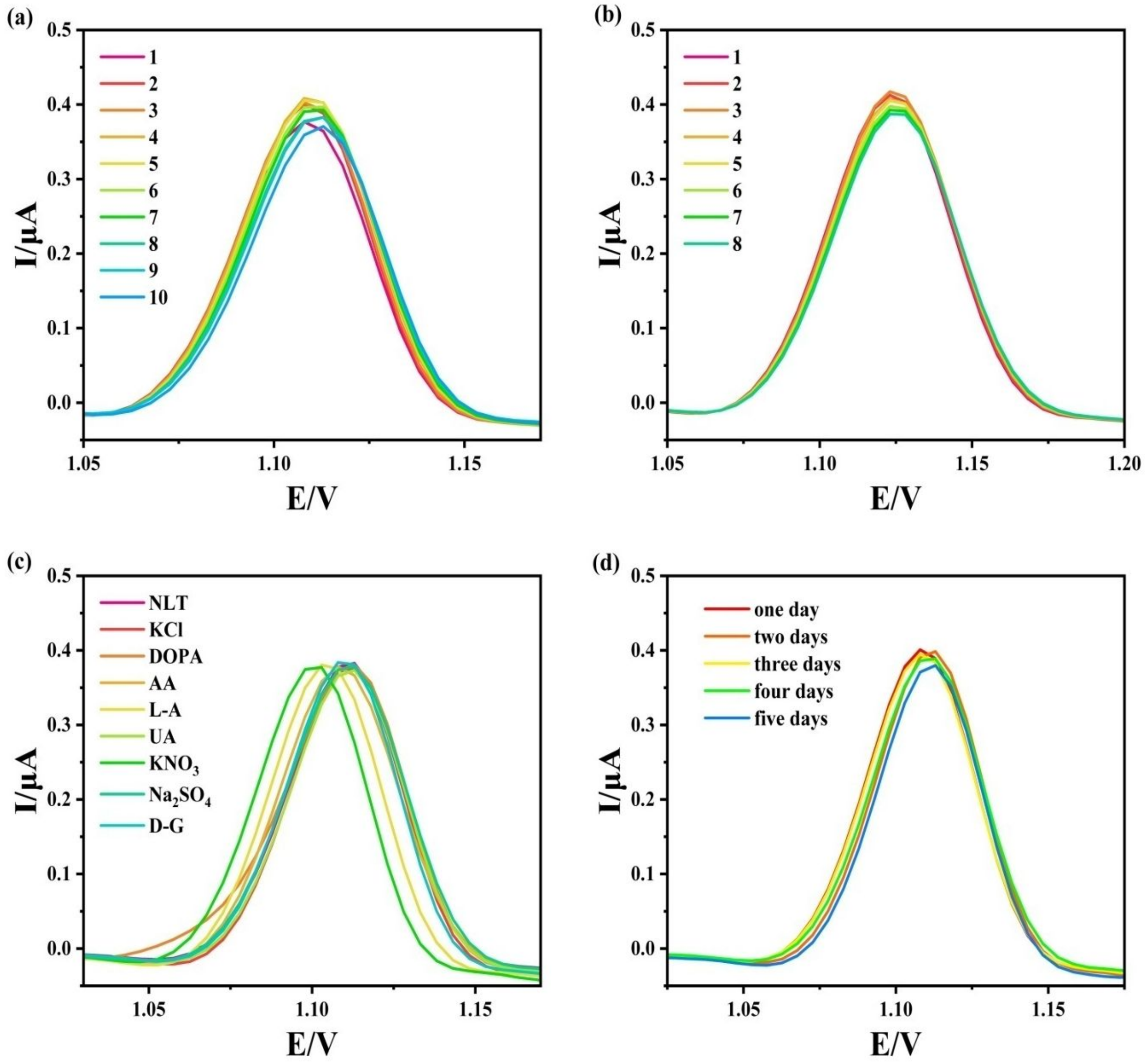

Figure S9. Repeatability (a), Reproducibility (b), Selectivity (c) and Stability (d) of 1.0 μM NLT at CoS@NAPC/GCE in PBS buffer (pH 2.0).

Table S2. Effect of differentinterferents on theoxidationcurrent of NLT (1.0 μM) at CoS@NAPC/GCE.

Interferent	NLT:Interferent	RSD(%)
**NLT**	1:100	3.75
**KCl**	1:1000	0.32
**DOPA**	1:100	3.40
**AA**	1:1000	2.48
**L-A**	1:1000	1.47
**UA**	1:1000	0.39
**KNO_3**	1:1000	0.28
**Na_2SO_4**	1:1000	0.17
**D-G**	1:1000	0.44
